# Are component endpoints equal? A preference study into the practice of composite endpoints in clinical trials

**DOI:** 10.1111/hex.12798

**Published:** 2018-08-14

**Authors:** Melissa C.W. Vaanholt, Marlies M. Kok, Clemens von Birgelen, Marieke G.M. Weernink, Janine A. van Til

**Affiliations:** ^1^ Department of Health Technology and Services Research MIRA – Institute for Biomedical Technology and Technical Medicine University of Twente Enschede The Netherlands; ^2^ Department of Cardiology Medisch Spectrum Twente Thoraxcentrum Twente Enschede The Netherlands

**Keywords:** best‐worst scaling, composite endpoints, coronary artery bypass grafting, coronary artery disease, patient preferences, percutaneous coronary intervention, revascularization, weighting procedure

## Abstract

**Objectives:**

To examine patients’ perspectives regarding composite endpoints and the utility patients put on possible adverse outcomes of revascularization procedures.

**Design:**

In the PRECORE study, a stated preference elicitation method *Best‐Worst Scaling* (BWS) was used to determine patient preference for 8 component endpoints (CEs): need for redo percutaneous coronary intervention (PCI) within 1 year, minor stroke with symptoms <24 hours, minor myocardial infarction (MI) with symptoms <3 months, recurrent angina pectoris, need for redo coronary artery bypass grafting (CABG) within 1 year, major MI causing permanent disability, major stroke causing permanent disability and death within 24 hours.

**Setting:**

A tertiary PCI/CABG centre.

**Participants:**

One hundred and sixty patients with coronary artery disease who underwent PCI or CABG.

**Main outcome measures:**

Importance weights (IWs).

**Results:**

Patients considered need for redo PCI within 1 year (IW: 0.008), minor stroke with symptoms <24 hours (IW: 0.017), minor MI with symptoms <3 months (IW: 0.027), need for redo CABG within 1 year (IW: 0.119), recurrent angina pectoris (IW: 0.300) and major MI causing permanent disability (IW: 0.726) less severe than death within 24 hours (IW: 1.000). Major stroke causing permanent disability was considered worse than death within 24 hours (IW: 1.209). Ranking of CEs and the relative values attributed to the CEs differed among subgroups based on gender, age and educational level.

**Conclusion:**

Patients attribute different weight to individual CEs. This has significant implications for the interpretation of clinical trial data.

## 1 INTRODUCTION

Over the past 40 years, many randomized clinical trials (RCTs) have used composite endpoints when comparing medical interventions.^1^, ^2^, ^3^, ^4^ These composite endpoints combine 2 or more clinically relevant endpoints, also known as the component endpoints (CEs), within a single outcome variable to measure clinical benefit of a treatment. The conclusions of RCTs rely on their primary endpoints, and thus, it is important to choose the most appropriate endpoints when designing clinical research.^5^ In recent years, medical care has significantly progressed for patients experiencing cardiovascular events, resulting in low mortality rates. Although death is still considered the primary outcome, it is often difficult for clinical researchers to identify differences in survival rates between the different treatment options.^6^, ^7^ To investigate the occurrence of an infrequent event, large sample sizes, as well as prolonged follow‐up, are needed, and costs go up.^8^ When several adverse events are combined in a composite endpoint, the occurrence of events will increase, thereby expanding the overall treatment effect, and reducing the required sample size and overall costs of cardiovascular trials.^9^, ^10^

Analytic approaches to composite endpoints generally assume that all underlying adverse events are of equal value. In practice, this assumption is rarely met, for instance: in some situations, the overall positive treatment effect may be related to “soft events” such as recurrent angina or redo revascularization as opposed to the clinically more relevant “hard” events such as major stroke or death.^11^, ^12^ This heterogeneity of effect among CE can result in too optimistic conclusions about the treatment effect and serious misinterpretations.^13^, ^14^, ^15^ One can account for these different effects by adjusting trial outcomes using “importance weights (values assigned to CE that reflect the relative importance of these CEs to patients)”. These “importance weights” are almost always derived through evaluations by an expert panel^11^, ^20^; however, previous research has shown that patient and expert preferences towards CE are different^21^ and thus cannot be considered equivalent. Therefore, the aim of this study was to examine patients’ perspectives regarding the use of composite endpoints in clinical trials and the importance they attach to possible unfavourable outcomes of treatment. In addition, we examined whether the obtained “importance weights” differed between subgroups based on clinical and demographic characteristics of our study population.

## 2 METHODS

### 2.1 Patient population

Between May 2016 and June 2016, the prospective, observational cohort PRECORE (PREference of COronary REvascularization) study was performed in a consecutive series of patients with coronary artery disease (CAD), who underwent revascularization procedures (either percutaneous coronary intervention [PCI] or coronary artery bypass graft [CABG]) at a tertiary centre for cardiovascular interventions (Thoraxcentrum Twente, Enschede, the Netherlands). PCI patients were included in this study 3‐4 hours post‐intervention. Patients who had a CABG procedure completed the survey on day 3 to 4 post‐intervention. Patients who underwent CABG plus a surgical intervention to correct cardiac valve disease were not included in this study. In addition, patients who were unable to perform the study task correctly due to the cognitive burden the study posed or due to a language barrier were excluded. The study protocol was submitted to the regional medical‐ethics committee (METC Twente, no. K16‐45), but was deemed exempt from formal medical ethical evaluation, because the study does not fall within the remit of the Medical Research Involving Human Subjects Act (WMO).^16^ All patients provided written informed consent, and all data were anonymized before analysis. The study complied with the Declaration of Helsinki. The literature provides no guidance to determine minimal required sample sizes for Best‐Worst Scaling (BWS) experiments. To determine the minimum sample size needed, we used a rule of thumb for conjoint analysis which states that estimate precision increases rapidly at sample sizes over 150 and flattens out at around 300 observations.^17^, ^18^ Taking into account the average number of patients undergoing a revascularization procedure throughout the 2‐month study period, we aimed at including at least 150 patients.

### 2.2 Patient preference survey

The original survey consisted of 4 different parts (Appendix S1). The PRECORE study started by asking patients to read the descriptions of the 8 CE examined in this study (Table 1). After patients read the descriptions, they were asked to answer 4 statements about whether they thought it was equally important to prevent 2 complications (*death* vs *disabling stroke; death* vs *disabling myocardial infarct* (*MI*); *death* vs *redo CABG;* and *disabling stroke* vs *disabling MI*). The statements examined whether or not patients weigh the CE equally. If patients answered at least one of these 4 statements with “yes, the avoidance of one of these 2 complications is more important to me, or they answered at least one of these 4 statements with “do not know,” the relative importance of each complication (CE) was examined by means of 6 Best‐Worst Scaling questions (BWS); the paragraph below explains this methodology. In addition to the preference elicitation questions, patients were asked for socio‐demographics and clinical profile and one final question was asked to directly examine their view on the use of composite endpoints (Appendix S3). The Web‐based survey was programmed using LimeSurvey^19^> and was intended for self‐completion on a tablet. However, if patients indicated that they needed more explanation or assistance in completing the survey, assistance was given. On average, it took patients 30 minutes to answer the complete survey.

**Table 1:** Attributes for the Best‐Worst Scaling case 1 choice‐questions

Treatment outcomes (Attributes)	Description to patients
Minor MI	You will experience a mild myocardial infarction of which the symptoms disappear within 3 mo after the initial myocardial infarction
Major MI	You will experience a large myocardial infarction causing permanent disability (ie tire more quickly, less physical capacity)
Minor stroke/TIA	You will experience a mild stroke of which the symptoms disappear within 24 h
Major stroke	You will experience a large stroke causing permanent disability (ie loss of function of an arm and/or leg)
Angina Pectoris	You will experience recurrent angina (ie sensation of chest pain, pressure or squeezing)
Redo CABG	You need to undergo a bypass surgery within 1 yr following your initial revascularization because of restenosis
Redo PCI	You need to undergo a PCI within 1 yr following your initial revascularization because of restenosis
Death	You will die within 24 h post‐intervention

### 2.3 Best‐worst scaling: A method for determining the relative importance of CE to patients

The attributes included in this study were determined in a stepwise manner, which subsequently included a literature review, expert review and individual interviews with patients. First, a list of attributes that describe possible unfavourable outcomes of revascularization was compiled based on previously published literature.^21^, ^22^, ^23^, ^24^, ^25^, ^26^ Second, this list of unfavourable outcomes was discussed within the steering committee of the research team (including 2 cardiologists with expertise in these interventions, and 2 senior health scientists for specialist methodological input). This expert review was conducted in order to (i) shorten the list of potential attributes and (ii) to ensure that the attributes were expected to be relevant for all patients who underwent a revascularization procedure. As a third step, 6 individual interviews were conducted with patients who underwent revascularization in order to ensure that (i) the most important attributes to patients were included and (ii) attribute descriptions were clear to patients. This process led to the inclusion of 8 attributes (Table 1).

The BWS method was used to determine the relative importance patients with CAD assign to the CE associated with coronary revascularization procedures.^27^, ^28^ BWS is based on the random utility theory, which assumes that a patient's relative preference for characteristic A over characteristic B is a function of the relative frequency with which A is chosen as better than, or preferred to, B.^29^, ^30^ This methodology was used, because it avoids and overcomes some of the limitations of rating‐ and ranking‐based measurement methods.^27^, ^28^ In BWS (case 1), respondents are asked to choose the best (eg least unfavourable) and worst items (eg most unfavourable) from a set of objects (ie adverse outcomes)^29^ (Appendix S2). By presenting several of these set of objects to multiple patients, and studying the probability of patients choosing one objects over the other, the relative desirability of treatment outcomes from the patients’ point of view (as a group) can be determined. The number of scenarios per patient was determined using the experimental design software Sawtooth 6.4.6. (Sequim, WA, USA). The most optimal design was a partial‐profile BWS case 1 design with 4 versions, 6 scenario‐questions per version, and 4 attributes per scenario.^17^

### 2.4 Statistical analysis

By use of IBM SPSS Statistics 23 (SPSS Inc., Chicago, IL, USA) and Stata version 14 (StataCorp, College Station, TX, USA), descriptive statistics were applied to get insights into the patient demographics and their perspectives regarding the use of CE in clinical trials (statement data). All analyses were 2‐tailed and applied on the aggregated sample level, as we were interested in overall group preferences. Best‐Minus‐Worst counts were calculated to study the distribution of scores. Best and worst counts represent the number of times an attribute level was chosen as best or as worst across all choice‐sets and respondents.^19^ By subtracting the total number of times it was chosen as worst from the total number of times an outcome was chosen as best, an initial ranking of all 8 attributes from best (ie least unfavourable) to worst (ie most unfavourable) can be determined. To account for the number of times the attribute was available in the BWS design, normalized scores were calculated; that is, the Best‐Minus‐Worst counts (B‐W counts) were divided by the sample size and the frequency that each attribute appeared in the design of the choice set. As it was chosen to use data on the aggregated sample level, no statistical analysis can be performed to analyse the potential significance of these B‐W counts. To explore potential heterogeneity in preferences between certain patient subgroups (gender, age, educational level, current revascularization procedure, previous revascularization experience and previous MI), several count analyses were performed. *P‐*values <.05 were considered statistically significant.

## 3 RESULTS

### 3.1 Patient inclusion

Of 176 patients contacted, 9 (5%) were excluded as they did not meet the inclusion criteria, 2 (1%) refused participation, and 5 (3%) were discharged too early to participate. A total of 160 patients met the eligibility criteria, agreed to be surveyed and were included in the PRECORE study. Some patients received hands‐on assistance by filling in the survey (n = 31, 19%) as they experienced physical constraint while filling in the survey. Another 6 (4%) patients received additional oral information and instructions after indicating a need for further assistance, and 9 (6%) patients received both hands‐on assistance and additional oral information and instructions. The surveys of 13 (8.1%) patients were returned with incomplete BWS‐data and could be not used for the analysis of the BWS‐data.

### 3.2 Patient characteristics

The patients’ sociodemographic and treatment‐related characteristics are presented in Table 2. Of the 160 patients included in this study, a total of 97 (61%) underwent PCI and 63 (39%) were treated by CABG. Patients were 67 ± 11 years old, and 120 (75%) were male. The majority of the respondents (n = 84, 52.5%) had a low level of education, and about a quarter (n = 40, 25%) was highly educated. A total of 86 (53.8%) patients had a previous MI, 9 (5.6%) a previous CABG, 45 (28.1%) a previous PCI and 5 (3.1%) experience with both PCI and CABG. A total of 105 patients (65.6%) had no history or previous coronary revascularization. Both patient groups had similar baseline profiles, but in line with clinical practice, significant differences were found between the PCI and CABG patients regarding the prevalence of *diabetes* (18.6% vs. 38.1%, *P *=* *.006, respectively) and *previous revascularization* (15.9% vs. 36.1%, *P *=* *.005, respectively).

**Table 2:** Baseline characteristics of the study population (n = 160)

	All patients (N = 160)	Revascularization procedure		*P*‐value
		CABG (n = 63, 39.4%)	PCI (n = 97, 60.6%)	
Sex				.707
Male	120 (75.0)	45 (71.4)	75 (77.3)	
Female	40 (25.0)	18 (28.6)	22 (22.7)	
Age, yr	67 (11.3)	68 (9.5)	66 (12.2)	.300
Younger age category (≤60 yr)	45 (28.1)	13 (20.6)	32 (33.0)	
Middle age category (61 ≤ 70 yr)	53 (33.1)	22 (34.9)	31 (32.0)	
High age category (70+ yr)	62 (38.8)	28 (44.4)	34 (35.1)	
Highest level of education				.144
Low education	84 (52.5)	29 (46.0)	55 (56.7)	
Middle education	36 (22.5)	13 (20.6)	23 (23.7)	
High education	40 (25.0)	21 (33.3)	19 (19.6)	
Risk factors				
Hypertension	76 (47.5)	33 (52.4)	43 (44.3)	.319
Hypercholesterolaemia	61 (38.1)	27 (42.9)	34 (35.1)	.986
Current smoker	36 (22.5)	14 (22.2)	22 (22.7)	.946
COPD	21 (13.1)	9 (14.3)	12 (12.4)	.726
Diabetes mellitus (any)	42 (26.3)	24 (38.1)	18 (18.6)	.006
Family history of CAD	39 (24.4)	13 (20.6)	26 (26.8)	.375
Previous MI	86 (53.8)	32 (50.8)	54 (55.7)	.546
Previous stroke	21 (13.1)	9 (14.3)	12 (12.4)	.726
Previous PCI^a^	45 (28.1)	10 (15.9)	35 (36.1)	.005*
Previous CABG^a^	9 (5.6)	2 (3.2)	7 (7.2)	.278

Data are n (%) or mean ± SD.

CABG, coronary artery bypass grafting; CAD, coronary artery disease; COPD, chronic obstructive pulmonary disease; MI, myocardial infarction; PCI, percutaneous coronary intervention.

a 5 patients have had previous CABG and previous PCI.

### 3.3 Statement data: Patients’ perspective regarding CE differ

A vast majority of patients (n = 129, 80.6%) stated that the common practice of weighing all CE equally is invalid, and more than half of patients (n = 94, 58.8%) indicated that it is more important to prevent a *major stroke causing permanent disability* than *death within 24 hours post‐intervention* (Table 3). Moreover, two‐thirds of the patients (n = 126, 78.8%) reported that it is more important to prevent *death within 24 hours post‐intention* than *redo CABG*. When patient's preferences of the CE were further analysed according to the patient's age, gender, previous MI or current type of revascularization procedure**—**no statistically significant differences were found (data not shown).

**Table 3:** Patient perspectives regarding the 4 statements (n = 160)

Statement	No. of patients	% of patients
Death vs major stroke	N = 160	100.0
Both complications are equally unfavourable	42	26.3
Avoidance of death is more important	26	16.3
Avoidance of major stroke is more important	85	53.3
Do not know	7	4.4
Death vs major MI	N = 160	100.0
Both complications are equally unfavourable	31	19.4
Avoidance of death is more important	94	58.8
Avoidance of major MI is more important	26	16.3
Do not know	9	5.6
Death vs redo CABG	N = 160	100.0
Both complications are equally unfavourable	21	13.1
Avoidance of death is more important	126	78.8
Avoidance of redo CABG is more important	6	3.8
Do not know	7	4.4
Major stroke vs major MI	N = 160	100.0
Both complications are equally unfavourable	38	23.8
Avoidance of major stroke is more important	104	65.0
Avoidance of major MI is more important	10	6.3

### 3.4 BWS‐data: Patients did not consider all CE equal

**Figure 1:**
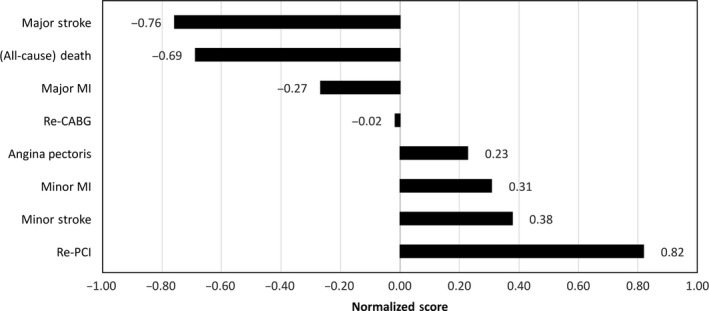
Standardized “best‐worst” scores for the 8 potential outcomes of revascularization. A total of 147 patients participated, each of whom chose best and worst attributes from 6 sets of 4 attributes each (4248 total choices). Standardized scores range from −1.0 to 1.0, where higher (positive) values indicate that a given attribute was chosen more often as best than worst, and were more likely to be preferred relative to the other attributes. A score of “0” means that an attribute was selected as best or worst an equal number of times.^15^ MI, myocardial infarction; re‐CABG, redo coronary artery bypass grafting within a year post‐intervention; Re‐PCI, redo percutaneous coronary intervention within a year post‐intervention

Systematic assessment by use of BWS showed that patients did not assign equal weights to all CE (Table 4). Figure 1 shows that patients considered the need to undergo a *redo PCI within one year post‐intervention* the least unfavourable (importance weight: 0.008). *Minor stroke with recovery within 24 hours* was the second least unfavourable CE (0.017), followed by (in the order of increasing importance to patients) *minor MI with recovery within 3 months* (0.027), *redo CABG* (0.119), *recurrent angina* (0.300) and *major MI causing permanent disability* (0.726). *Major stroke causing permanent disability* was considered worse than *death* and all other CE (1.209). The preference data of patient subgroups are shown in Table 4. The rank orders of most subgroups resemble the average estimate, except that highly educated patients (n = 36), females (n = 29), and elderly patients (n = 57) place greater emphasis on avoiding *minor MI* than *recurrent angina*. Furthermore, a notable difference was that highly educated patients (n = 36) valued *death* the most unfavourable outcome of this subset of outcomes, while the overall study population “preferred” *death* over *disabling stroke*.

**Table 4:** Estimate of subjective priority scores for attributes and rank order variations using the count analysis method (n = 147)

Attribute	Overall patient population (n = 147)			Normalized score	Rank	Patient subgroups		
						Education_High (n=36)Gender_female (n=29)Age_High (>70 years old, n=57)		
	No. of times chosen					Rank order variations		
	Total best	Total worst	B‐W			Rank	Rank	Rank
Re‐PCI	364	2	362	0.82	1	1	1	1
Minor stroke	173	4	169	0.38	2	2^a^	2	2
Minor MI	143	8	135	0.31	3	4	4	4
Angina pectoris	118	17	101	0.23	4	2^a^	3	3
Re‐CABG	74	81	−7	−0.02	5	5	5	5
Major MI	3	122	−119	−0.27	6	6	6	6
(all‐cause) Death	4	309	−305	−0.69	7	8	7	7
Major stroke	3	339	−336	−0.76	8	7	8	8

MI, myocardial infarction; re‐CABG, redo coronary artery bypass grafting within a year post‐intervention; Re‐PCI, redo percutaneous coronary intervention within a year post‐intervention.

*Note:* ^a^Attributes with the same rank order have equal B‐W Counts.

## 4 DISCUSSION

The PRECORE study examined the patients’ perspective regarding the use of composite endpoints and the utility patients put on possible unfavourable outcomes of treatment. While it is common practice in clinical trials to weigh individual adverse outcomes of medical treatment equally, a vast majority of patients considers this approach as invalid. Our study shows that patients place greater emphasis on avoiding “hard” cardiovascular events (*death, major MI, major stroke*) than “soft” events, such as *redo revascularization* (both PCI and CABG), *minor stroke*,* minor MI* and *recurrence of angina pectoris*. In addition, more than half of the patients stated that the avoidance of a *major stroke* is more important than the avoidance of *death,* suggesting that patients fear a loss of their mobility and independence most of all. Our results corroborate the findings of Ahmad et al^26^ and Stolker et al^21^ who described that patients considered disabling stroke worse than death. In addition, patients place greater emphasis on avoiding a *redo CABG surgery*, as compared to a *redo PCI procedure*, and do assign different weights to CE according to severity (major/minor event). These results are interesting as most current ongoing RCTs do not categorize their clinical outcomes according to event severity or type of *redo revascularization procedure* (PCI vs. CABG). In addition, although the ranking of CE was the same for patients of the PCI vs the CABG group, patients of the CABG group placed greater emphasis on the avoidance of *a redo CABG procedure* than PCI patients. One may speculate that knowledge about the full impact of this surgical procedure instigated patients of the CABG group to place greater relative importance on *redo CABG* than patients of the PCI group did.^34^, ^35^ The use of composite endpoints to compare competing interventions is only a valid reflection of the relative value of different interventions if each CE is viewed as equally important to patients. The current study and previous research in this field suggest that this is not the case.^11^, ^24^ In accordance with previous recommendations, we therefore recommend using “weighted” CE, in which individual CEs a valued relative to one another.^11^, ^20^, ^22^, ^23^, ^25^, ^26^, ^36^ Prior efforts to weigh these CE often assumed that patients, doctors and other experts would assign similar values to individual CEs; however, the study of Stolker et al.^21^ showed that this is not the case. Where patients were most concerned about reducing MI or stroke, clinical trials placed greater emphasis on avoiding death.^21^ Consequently, we advise an alternative method that in concept is similar to the “weighted effect measure” methodology as stated by Armstrong et al.^20^ In that methodology, the authors allocated weights that reflect the relative severity of individual CEs to patients; and the weights were determined through a clinician‐investigator Delphi panel.^21^ However, instead of experts assigning weights to CE, we suggest incorporating patient preferences in the evaluation of CEs. In addition, it is important to reach agreement on which method is most appropriate to measure patient preferences for adverse outcomes of treatment, such that normalized “importance weights” can be determined, and applied to raw trial data. Meanwhile, existing clinical trial data should be carefully interpreted, as these “non‐weighted” data could be misleading.

### 4.1 Strengths and limitations

This study has both strengths and limitations. To the best of our knowledge, this is one of the first studies that quantified the differences patients attributed to each CE using a choice‐based method and to study whether or not patients agree with the scientific practice to combine multiple CE into one composite endpoint. The quantitative nature of this prospective, observational cohort study enables us to obtain insights into the distribution of preferences and the possible differences in these preferences between subgroups of patients. In addition, CE in this study are categorized according to severity and type of procedure (ie major/minor MI or stroke, and redo PCI/CABG). The present study has some limitations. First, we cannot exclude that the results of this single‐centre study might be influenced by local clinical, geographical and socioeconomic factors, which limits generalization of the findings. Second, we cannot exclude that the views and priorities of patients, their physician and their family members, and their prior (treatment) experiences may have driven preferences in this context. For instance, the obtained stated preferences reflect patients who had just undergone PCI or CABG. It might be that post‐interventional preferences differ from preferences before the intervention, as patients may be influenced by the new experience. A prior study by Kipp et al.^24^ among patients with established CAD or who are at high risk for CAD, however, demonstrated that patient history of PCI and CABG did not influence their choice of mv‐PCI or CABG across hypothetical risk scenarios. The preferences of patients who previously had both PCI and CABG were similar to those with no history of these procedures (OR = 1.02, 95% CI: 0.28, 3.73).^24^ Third, higher educated patients place greater emphasis on avoiding minor MI and less emphasis on avoiding a major stroke compared with the overall study population. Prior studies have shown that patient preferences can be influenced by the wording of attributes, or by health literacy and educational level.^37^ Whether differences in these preferences are actually due to varying preferences, our somewhat broad brush CE descriptions, or might be better explained by varying levels of understanding the true ramifications of the different health outcomes should be further investigated.

## 5 CONCLUSIONS

The majority of patients in the PRECORE study indicated that they do not agree with the common practice of weighing clinical endpoints equally. Patients considered “hard” cardiovascular events significantly more unfavourable than “soft” events. One of 2 patients stated to be more worried about permanent stroke causing disability than death, suggesting that many patients fear a loss of mobility and independence above death. The findings of this study demonstrate that the current practice of most clinical trials does not reflect patients’ preference and encourage a shift in thinking that may lead to importance weight‐adjusted composite endpoints for clinical trials.

## CONFLICT OF INTEREST

All authors have completed the ICMJE uniform disclosure form at http://www.icmje.org/coi_disclosure.pdf and declare: CvB reported institutional research grants provided by Biotronik, Boston Scientific and Medtronic. All other authors declared that they have no conflict of interest.

## AUTHOR CONTRIBUTIONS

CvB, MK, JvT and MW conceptualized the original study proposal. All authors contributed substantially to the design of the work; the acquisition, analysis and interpretation of data for the work; and the drafting the work or revising the manuscript critically for important intellectual content. MV searched the literature, collected the data, analysed the data and wrote the first draft of the manuscript. MW provided statistical expertise. As principal investigator, MV had full access to all of the data in the study, and takes responsibility for the integrity of the data and the accuracy of the data analysis. She is the guarantor.

## ETHICAL APPROVAL

The PRECORE study received approval from a medical ethical committee, and participants gave written informed consent.

## DATA SHARING STATEMENT

No additional data available.

## TRANSPARENCY STATEMENT

The lead author affirms that the manuscript is an honest, accurate and transparent account of the study being reported; that no important aspects of the study have been omitted; and that any discrepancies from the study as planned have been explained. This is an open‐access article distributed in accordance with the Creative Commons Attribution NonCommercial (CC BY‐NC 4.0) licence, which permits others to distribute, remix, adapt, build upon this work non‐commercially, and license their derivative works on different terms, provided the original work is properly cited and the use is non‐commercial. See: http://creativecommons.org/licenses/by-nc/4.0/.

## LICENCE TO PUBLICATION

I [C. von Birgelen, MD PhD] The Corresponding Author of this article contained within the original manuscript which includes any diagrams & photographs within and any related or stand alone film submitted (the Contribution”) has the right to grant on behalf of all authors and does grant on behalf of all authors, a licence to the BMJ Publishing Group Ltd and its licencees, to permit this Contribution (if accepted) to be published in the BMJ and any other BMJ Group products and to exploit all subsidiary rights, as set out in our licence set out at: http://www.bmj.com/about-bmj/resources-authors/forms-policies-and-checklists/copyright-open-access-and-permission-reuse.
